# Addressing harm in moral case deliberation: the views and experiences of facilitators

**DOI:** 10.1186/s12910-020-0450-2

**Published:** 2020-01-30

**Authors:** Benita Spronk, Guy Widdershoven, Hans Alma

**Affiliations:** 1 Amsterdam UMC, Vrije Universiteit Amsterdam, De Boelelaan 1117, Postbus 7057, 1007 MB Amsterdam, Netherlands; 2 Department of Medical Humanities, Amsterdam UMC, Vrije Universiteit Amsterdam, De Boelelaan 1089 a, 1081 HV Amsterdam, Netherlands; 3 Department of Philosophy and Ethics, VUB (Vrije Universiteit Brussel), Pleinlaan 2, 1050 Brussel, Belgium

**Keywords:** Tragedy, Moral case deliberation, Decision-making, Harm, Repair

## Abstract

**Background:**

In healthcare practice, care providers are confronted with decisions they have to make, directly affecting patients and inevitably harmful. These decisions are tragic by nature. This study investigates the role of Moral Case Deliberation (MCD) in dealing with tragic situations. In MCD, caregivers reflect on real-life dilemmas, involving a choice between two ethical claims, both resulting in moral damage and harm. One element of the reflection process is making explicit the harm involved in the choice. How harmful are our decisions? We investigated how facilitators of MCD experience the importance of addressing harm in MCD and what participants learn from reflecting on harm.

**Methods:**

The study was qualitative, focusing on the views and experiences of the facilitators of MCD. Semi-structured interviews (*N* = 12) were conducted with facilitators of MCD. The research focuses on the subjective experiences of facilitators. Grounded Theory was used for analysis.

**Results:**

The results show two main categories. The first concerns the awareness of tragedy. Within this category, five themes were discerned: making explicit that there is no solution, visualizing consequences, uncovering pain, focusing on emotion, and exploring perspectives on harm. The second category concerns the support for healthcare professionals in dealing with the tragedy of the choices they face. In this category, five themes came forward: acknowledging, offering comfort, managing harm, consideration through dialogue and repairing harm.

**Conclusion:**

Our study shows that addressing harm in MCD in tragic situations provides an important moral learning opportunity for participants. By formulating and becoming aware of harm, MCD aids healthcare professionals in the task they are faced with, namely making difficult and painful choices. MCD helps healthcare professionals to repair moral damage, making clear at the same time that harm cannot be undone.

## Background

Healthcare providers are often confronted with tragic situations. Examples are: Should we stop treating a young woman who has suffered a severe cerebral haemorrhage and slipped into a coma? Should we keep a seriously ill baby in the hospital who has been there for months with no hope of recovery, or discharge the baby, so that it will die at home? Can a scheduled operation be postponed a third time? In such situations, healthcare professionals face a dilemma. The choice they make will inevitably be at the expense of the alternative – every choice causes harm.

In healthcare practice, Clinical Ethics Support (CES) is available to support professionals in making difficult ethical decisions. Moral Case Deliberation (MCD) is one of the instruments applied in CES [1–4]. The aim of MCD is not to provide expert advice, but to facilitate reflection [5, 6]. During MCD, healthcare professionals involved in a concrete situation jointly investigate moral issues in healthcare [7]. Studies have been conducted on goals [1], practice [5], implementation and impact of MCD [8, 9]. However, little research has been conducted on the role of MCD in dealing with tragic situations [10]. An important element in MCD is making explicit the moral damage and harm that a choice entails. Identifying the harm in a moral dilemma is a fixed part of deliberations run using the dilemma method. By identifying the potential harm, the tragedy of the case is put ‘under the magnifying glass’. Little attention however is given to what the professional has to give up, when a course of action is chosen in a dilemma. The research question addressed in this paper is: How can MCD support healthcare professionals in tragic situations by addressing this harm?

In our concept of harm we follow Nussbaum [11, 12]. Harm concerns the moral damage that cannot be avoided or resolved. This differs from the four principles approach of Beauchamp and Childress including the non-maleficence principle ‘one ought not to inflict evil or harm’ [13], often used in Ethics Consultation. In Ethics Consultation harm is a topic of reflection in weighing up beneficial and non-beneficial effects [14, 15]. Doing so requires balancing the outcomes and choosing the best solution [16]. Following Nussbaum, whatever option one chooses, a fundamental moral damage remains, which cannot be resolved, but needs to be acknowledged. This notion of harm is addressed in this paper.

Literature on tragedy often focuses on experiences of patients living with a serious illness, trauma or disability [17–22]. Sometimes, literature addresses the burden experienced by healthcare professionals due to the difficult decisions they face, and their consequences [23, 24]. In this article we examine the role of MCD in supporting healthcare providers who are confronted with tragic choices.

MCD sessions are guided by trained facilitators, whose experiences with MCD constitute a source of knowledge. We therefore aim to tap into their views and experiences of addressing harm. We will also examine whether and, if so, how making harm explicit supports professionals in dealing with tragedy.

We will start by defining the concept of tragedy based on the literature, followed by a description of the research method applied and the results obtained. The discussion will reflect on the results, after which a conclusion will be provided.

### The concept of tragedy

Tragedy relates to the vulnerability of life [25]. Tragic situations require choices that involve suffering and death. These choices are often accompanied by feelings of powerlessness, and entail threats to human dignity [10]. If there is any place where such situations abound, it is in hospitals.

Care providers often have to take decisions that will result in harm no matter what they choose. Nussbaum draws a distinction between situations that can be resolved by a cost-benefit analysis, and those that involve deciding what to give up. She refers to the latter as ‘the tragic question’ [11].

According to Nussbaum, the greatest challenge lies in the moral choices that people are thus forced to make. These she refers to as ‘the tragic conflict’, and they are considered tragic because they force action in situations where each available choice is accompanied by inevitable moral objections. ‘In such cases we see a wrong action committed without any direct physical compulsion and in full knowledge of its nature, by a person whose ethical character or commitments would otherwise dispose him to reject the act.’ [12]. Nussbaum argues it can be beneficial to reflect on such situations, as they force us to consider what constitutes ‘personal goodness’. Nussbaum has studied the Greek tragedies extensively. As an example of a tragic conflict, she cites Agamemnon who must choose between abandoning his expedition to Troy, or sacrificing his daughter. The gods put him in this situation, and there is no escape without acquiring blame [26]. However, Agamemnon supposes that there is a proper course of action – the lesser of the two evils [27] – and he does not see the harm caused by choosing that course. The chorus reproaches Agamemnon for not acknowledging the severity of his deeds, for showing no remorse, and for accepting them as pious and just [28]. According to Nussbaum, we must acknowledge the harm that will always exist on both sides of the dilemma.

In summary, tragedy occurs when both choices in a dilemma have morally bad consequences. Treatment providers are expected to make these types of decisions when treating vulnerable patients who face the prospect of illness, suffering and death. These choices affect professionals in their working life and their personal values. While we do not want to trivialise the impact of tragic situations on patients, this article will focus on the tragic conflicts faced by healthcare providers.

### Moral case deliberation and the dilemma method

Moral Case Deliberation (MCD) is a structured method for investigating a moral issue experienced by participants in practice. It is guided by a trained facilitator. MCD participants are often healthcare professionals (doctors, nurses, paramedics), but can also include managers, family members or patients themselves. The principal element is a case presented by one of the participants. The case must entail a concrete, personal experience from the past or present – not a hypothetical situation [5].

The main aim of MCD is not to arrive at a solution, but to jointly explore the ethical question at hand and thus encourage critical reflection on the values associated with the concrete facts of the case [5]. The reflection is brought about by dialogue. Dialogue is a teaching method involving the exchange of various perspectives in order to arrive at a ‘fusion of horizons’ [29] among the participants. The purpose behind exchanging perspectives is to search for common ground between one’s own and others’ experiential horizons, in order to understand one another better and get a richer view of the situation.

To structure the discussion, the facilitator uses a specific conversation method. A well-known method is the dilemma method [5, 30], which formulates the ethical issue as a dilemma, presented as two mutually-exclusive treatment options. The dilemma method entails the following steps: 1. Introduction; 2. Presentation of the case; 3. Formulating the moral question and the dilemma; 4. Clarification in order to place oneself in the situation of the case presenter; 5. Analysing the case in terms of perspectives, values and norms; 6. Looking for alternatives; 7. Making an individual choice and making explicit one’s considerations; 8. Dialogical inquiry; 9. Conclusion; and 10. Evaluation [5].

In this method, moral damage or harm is explicitly addressed in two of the steps: first in step 3, when the dilemma is formulated and the negative moral consequences of both options are made explicit; and second in step 7, when participants are asked to make an individual choice and to specify how they might repair the harm related to that choice. The dilemma method is thus in line with Nussbaum’s concept of tragedy discussed above. Other methods can also be applied to facilitate MCD [31]. In such methods, the harm associated with the moral decision is not always explicitly addressed.

## Method

### Data collection

Semi-structured interviews were held by the first author with MCD facilitators, who were asked to give examples of MCD sessions run by themselves that involved a tragic situation, and to explain them briefly. Facilitators using the dilemma method were asked about the role of addressing harm in the MCD. Respondents who do not use the dilemma method were asked how the aspect of harm is brought up in their own method. They were also asked for their opinions on the explicit identification of harm in the dilemma method.

The following criteria were used to select facilitators:

- Extensive experience in MCD facilitation (a minimum of one year’s experience as a facilitator);
- Currently working in healthcare (a hospital, psychiatry); and
- A representative distribution in gender, age, professional backgrounds and fields of operation.

Twelve facilitators were interviewed (six male and six female) at their workplace or at their home. A procedure of purposive selection was followed. As the respondents were known for their experience in MCD, they were asked by email to participate. No one refused. The respondents included two clinical ethicists, three spiritual counsellors, three medical specialists, one paramedic, two healthcare managers, and one nurse manager, thus spanning a wide range of educational backgrounds. The respondents worked as facilitators with various groups. Six worked in hospitals, three in mental healthcare, and three in both. The age of the respondents ranged between 30 and 68 years. The facilitators used a range of MCD methods: eight used the dilemma method, next to other methods, four only used other methods. These methods entail value clarification and the Socratic dialogue. Although these methods are similar in reflecting on an ethical case, they do not take the ethical dilemma as a starting point for the deliberation.

After obtaining the respondents’ consent, all interviews were recorded, transcribed, and de-identified by the first author and an assistant. The VU University Medical Centre’s Medical Research Ethics Committee declared the study did not fall under the Medical Research (with Human Subjects) Act (*WMO*), as no interventions were performed.

### Data analysis

The study aimed to identify the key elements of addressing harm as part of MCD, based on the facilitators’ personal experiences. The facilitators’ experiences were defined as broadly and openly as possible. To this end, we made use of the Grounded Theory approach as developed by Charmaz [32].

Data analysis was carried out in three stages. The first stage involved open coding: the first two interviews were coded independently by two researchers, and the results compared in order to establish inter-rater reliability. The results were discussed by all three authors. Based on this process, the topic list for the next interviews was refined. The next two interviews were coded by the first author, after which the three authors reflected on the resulting coding tree, in order to foster validity. The first researcher then conducted another eight interviews, two of which were also coded by a research assistant to again establish inter-rater reliability.

During the second stage, focused coding, eight interviews were analysed, and codes were clustered into overlapping themes. The results were discussed by the first two authors deciding on the best phrasing of the themes. This produced ten over-arching themes, formulated as ‘gerunds’ in accordance with Tweed and Charmaz [32, 33]. Gerund-based coding ensures a focus on actions rather than concepts, retaining a closer connection to the data (e.g. ‘visualising consequences’ instead of just ‘consequences’). This approach suited our study, as we sought to investigate how harm was actually addressed in MCD practice.

The third phase – axial coding – examined the relationships among and patterns between the various themes, after which the over-arching themes were refined and the final categories formulated. After the full analysis of eight interviews, theoretical saturation was reached. We analysed the other four interviews, with participants from various backgrounds. In the analysis no new themes were found.

## Results

The section below describes the categories and themes identified. They derive from multiple cases. Examples are forced treatment versus private integrity, the request of relatives to continue treatment versus the professional account that prolonging treatment is medically useless and adds suffering (and vice versa), stopping or continuing treatment in the neonatology ward and rights of potential parents versus the rights of the child in fertility treatment. A summary of the categories and themes is given in Table 1.

**Table 1:** Summary of the key elements in addressing harm

Category	Theme
1. Awareness of tragedy	Realising that there is no solution
	Visualising consequences
	Uncovering pain
	Focusing on emotion
	Exploring perspectives on harm
2. Dealing with tragedy	Acknowledging tragedy
	Offering comfort
	Managing harm
	Consideration through dialogue
	Repairing the harm

### Awareness of tragedy

The first category is the awareness of tragedy, and covers multiple themes.

#### Realising that there is no solution

In the first place, identifying harm can help MCD participants realise that there is no solution. This is exemplified in the following quote:

*I do often bring it up myself... and it does help... It helps because sometimes people are still trying to avoid harm completely, they are looking for the best solution. And it just helps to actually confront the reality, that it’s just not going to happen! (Interview 5)*

#### Visualising consequences

In the second place, addressing harm makes the consequences of both options visible, and shows what is at stake in the dilemma.

*I think that formulating the harm really helps drive home the implications and consequences of the treatment options to the participants. So it always helps, because it reveals what is at stake. (Interview 1)*

*One of the most important things in her case, and the MCD that really helped her, was creating the overview of the negative consequences of her decision. [...] She really hadn’t considered any of the adverse effects or the potential harm, she just thought she could deal with the situation. It was an important thing for her to realise. (Interview 7)*

One respondent mentioned a downside of focusing on negative consequences; according to this respondent it can sometimes be helpful to identify the main benefits of each option, instead of concentrating on the negative consequences.

*People sometimes ask why I never list the positive aspects of each side in the dilemma. ‘Good question’ I think! But we do it this way anyway, because of the tragic character of the situation. But sometimes I also think to myself, yeah, in certain situations it could also be really useful. So instead of the harm on each side of the dilemma, [...] we could look at what would be the best reason to go for option A, or what the greatest benefit would be. (Interview 5)*

A limitation of visualizing consequences is that they are not yet real during the MCD, but only surface later.

*And at that moment, the biggest fallout resulting from the decision is invisible to the participants in the MCD. Because either the people trying to get pregnant don’t get help, [...] we refuse to start treatment. So that’s one potential form of harm. But we could also agree to treat them, and then their child could have some serious condition, but we wouldn't see that either if it did happen. (Interview 9)*

#### Uncovering pain

Addressing harm exposes the pain involved in the dilemma:

*We find it hard to make a choice, because we see that choosing one thing will bring about consequences that are also painful and difficult. (Interview 6)*

*It’s a very useful way to talk about harm, for parents too. [...] Because in a situation like this, they are of course dealing with a huge amount of pain. (Interview 2)*

A possible downside noted by one respondent (who does not use the dilemma method) is that the pain thus uncovered is magnified. For this reason, this respondent explores the moral values of those involved, without explicitly asking for the harm.

*No, we don’t insist on people listing the harm. [...] What we do is explore the values of those with a moral stake in the situation. (Interview 6)*

This respondent associates harm with waddling in despair.

*So should we explicitly dig and ask, what kinds of horrible things will happen if we do such and such? That’s not how I work! No way. (Interview 6)*

#### Focusing on emotion

Addressing harm also entails focusing on emotion. Identifying harm shows how people are moved by the issue.

*We look at A and B, then formulate the dilemma, and then the harm on both sides. So it’s usually inherent to the method, in which case it is often expressed emotionally. If someone starts off saying, no I can't, I can't simply stop treatment if there are still options available (talking about the harm of option A) or whatever it is, then you see the harm emerging very clearly. (Interview 5)*

A downside noted by some respondents is that the focus on emotion through identifying harm can stand in the way of reasoning and deliberation.

*And once you find yourself in a tragic situation, it’s hard to keep a clear head. And why is that? Because you get so caught up in your visceral and emotional responses that you can’t separate yourself from them anymore, sometimes not for years! That’s how tragic these things can get. But the shift, there should be a shift towards: ‘What do I actually think?’ Why do I keep wallowing in the tragedy? (Interview 7)*

*We’re only human and these things touch us deeply. And that, I think, is the important thing about our moral sensitivity, that it is touched, because that’s what the facilitator is asking for. [...] You mustn’t shut out your emotions, but emotion shouldn’t be the primary focus. (Interview 4)*

#### Exploring perspectives on harm

The fifth theme involves exploring perspectives on harm. Asking participants to make harm explicit during MCD creates insight in other MCD participants’ views on harm.

*Well, I do think it helps clarify things for the participants, and helps them understand what other people see as damaging. (Interview 9)*

*Yes, I ask them about it (the harm) point-blank, because I think it’s necessary to achieve the second goal of MCD, which is understanding each other better. (Interview 11)*

The various perspectives of the care provider, patient and family on harm are all included in the discussion.

*When asking the question ‘what constitutes proper care?’ I think it’s really critical to look at the resulting harm. Like if we decide on a certain course of action, what is it we’re trying to avoid? And that might be our view as health professionals, but what do patients think? Or their families? I think it’s very important. (Interview 10)*

### Dealing with tragedy

The second category relates to how discussing harm can help those involved to deal with tragedy.

#### Acknowledgements

In the first place, discussing harm can help to deal with tragedy through the acknowledgement of harm. In this context, attention to unresolvable issues and acknowledgement of long-term harm are important.

*The loss or lingering remains of harm... sometimes it might even actually be helpful to spend some time thinking about that, about the things you can’t resolve, the lasting effects, and to acknowledge them. (Interview 1)*

*Approaching and standing face-to-face with tragedy can make us feel very lonely. But the simple acknowledgement of ‘it is what it is’ can be tremendously liberating – and an enormous relief, because of the feeling of acceptance. There’s no longer a need to fight it, to oppose it or to resolve it. The first step is to ‘acknowledge what it is for a moment'. (Interview 4)*

#### Offering comfort

Secondly, discussing harm provides comfort. Participants relate to the harm on both sides of the dilemma, which is why it is unresolvable. The facilitator’s job is to be attentive of this. Certain things in life cannot be resolved or repaired, and making harm explicit can offer comfort or relief.

*I think we all sometimes feel tempted to try to resolve a situation without causing any harm, whereas if you realise it's simply not possible, that you’ll need to weigh things up, that there will inevitably be harm somewhere... it gives you peace, more peace of mind, more comfort, something like that. (Interview 5)*

#### Management

Thirdly, MCD can equip and empower health professionals by making them reflect on possibilities to manage harm resulting from their choice in the dilemma.

First of all, exploring various perspectives on harm can help.

*Of course, this case involved multiple types of harm, yes. And now that you mention it, there was a very nice turning point [ … ], when one of the doctors came out with a real eye-opener, he said: ‘ who are we to say (to the patient) ‘too bad, you have yourself to blame, now here you are again after wasting six months’. Perhaps to him those six months weren’t wasted? Perhaps they were worthwhile. (Interview 11)*

Secondly, MCD can help to manage harm through reflection on the relationship between harm and living a good life, which can lead to the realisation that there is no such thing as a perfect life.

*Suppose you’ve found the good life, a medically perfect life, you’re home and housed... then what? That’s when I asked all those critical questions I mentioned to the group. (Interview 11)*

Thirdly, making harm explicit may equip and empower health professionals with management strategies. The investigation of tragic cases in MCD focuses on the question: What lies within my power? What resources can you draw on to help you realise it?

*So at some point we might decide on something particularly horrible – what should we do? It’s the lesser of two evils. The first thing I do is look at what you can draw on to help you. Have you been in a similar situation before? What worked then? Do you maybe know someone who can help you? And how can we check that it will work out? It’s about giving people strategies, and empowering them. (Interview 6)*

*Plus, I see MCD as a way of giving mainly health professionals, but others too, ways of managing and dealing with the type of tragedy that is inherent to our practical reality as well as they can. (Interview 4)*

#### Consideration through dialogue

In the fourth place, harm can be considered in dialogue. Dialogue is important when seeking to establish why a certain course of action should be pursued. Such a dialogue can take place among colleagues, as evinced by the following interview excerpt:

*Interviewer: So the weighing up is achieved through...*

*RES: Dialogue!*

*Interviewer: Through discussion in dialogue with one another?*

*RES: So it’s not like I say, how much is this worth and how much is that worth, and we do a little addition and subtraction and then we’re finished, no. (Interview 6).*

The dialogue may also involve a patient and/or their family.

*We decided to turn off the respirator of a young woman who had suffered cerebral haemorrhaging. The harm in that case was the family’s extreme emotional response, because as you can imagine, ceasing treatment based entirely on medical grounds is pointless. [ … ] And the way to limit the harm in that case was actually to enter into dialogue with them, to start a conversation and to give your reasons for making your decision and why there is no point in continuing with treatment. (Interview 8)*

In this dialogue, weighing harm is not a matter of quantity – it is a personal consideration that others might make differently.

*I think it’s important to understand that it’s not about quantity, it’s not like this option is less damaging than that option so that’s automatically the right answer. I also think it’s important to realise that people weigh things up in their own way, and that for others the balance might fall differently. (Interview 5)*

Respondents state that weighing harm is not a mathematical operation. Discussing the harm through MCD helps establish a bond among the participants.

*There are times when you think... this is just tragic. There’s no way out. It’s actually quite heartbreaking, and a good thing that we all feel it together. That’s the bond you feel as part of a tragedy. [...] Then something changes during the session, something that I believe is very powerful. In other words, I think using a kind of technical formula – ‘we’ve got some pros and cons on the scales so this is what we’re doing’ – would be a missed opportunity. (Interview 2)*

The subject of limiting harm is also addressed when searching for a middle ground.

*I think it [the harm] is automatically included in weighing things up, perhaps also in looking at resolvability, or opportunities for limiting the harm. A week or two ago we ran an MCD here. [...] And ultimately we arrived at the same middle ground. Well, we still needed to check a couple of things. But all the doctors and nurses said ‘we’re doing A, provided we can be certain’ and all analysts said ‘we’re doing B, unless there is evidence to the contrary.’ (Interview 9)*

#### Repairing harm

Finally, addressing harm as part of MCD helps the participants to deal with tragedy by looking at whether harm can be repaired.

*That’s a very important part of everything too, of course, that whichever option you choose, A or B (and sometimes there’s also C or D), there will always be drawbacks. It helps to get them out in the open and to look at what can be salvaged, so to speak, at what extra things can be done to limit the harm. (Interview 3)*

*That’s the unique thing about the method. MCD goes like this: you look at the situation, and make a decision between A or B. But any decision you make will always automatically cause harm. That’s the tragic thing about dilemmas, there will always be harm, no matter what you do. But you can also look at how to keep it to a minimum. (Interview 8)*

One downside mentioned is the fact that it is not always possible to repair harm. Some of the respondents pay special attention during MCD on specifically identifying this type of harm, which cannot be reduced or eliminated.

*So MCD is all about deciding between two evils. You identify the harm and then ultimately weigh things up: what the plan is, why that plan was chosen and how the harm can be reduced. But the harm that will always be there, that never goes away, is never listed separately, and that is an element I would like to add. (Interview1)*

## Discussion

Using the Grounded Theory approach, this study investigated the role of addressing harm as a part of MCD in discussing tragic situations.

Under the awareness category, the results first of all show that discussing harm in MCD can help healthcare professionals to realise that there is no ideal solution, a result that is in keeping with Nussbaum’s viewpoint. Care providers must make a choice between two ethical claims, both of which result in loss. This can be explained further by referring to the example of the choice between stopping treatment and letting a young woman die or keeping her alive in a condition of unconsciousness, mentioned in the introduction. In such a situation, care providers experience the limits of their professional competence. The harm they have to deal with is not only the harm for the patient, but also the harm for the care professional feeling responsible for his choice. This differs from an (avoidable) error or mistake and concerns harm on a fundamental level.

Secondly, the results showed that putting the harm into words can help to visualise the consequences of both options in the dilemma, and to take stock of the negative consequences of a decision. Some downsides were also named: in addition to identifying the harm caused, respondents feel it would also be worthwhile to focus on the benefits of each decision. The second downside is that the harm can only be estimated and not fully assessed during the discussion. The facilitator can assist in this regard, by using the perspectives of all participants to examine the severity of the harm for each individual as effectively as possible, and to envisage the future impact and consequences of any particular decision together as a group.

Thirdly, putting harm into words helps to uncover the pain involved in the moral dilemma – both the pain experienced by patients and their loved ones, and agonising decisions that must be made by health professionals. This is reminiscent of ‘moral injury’ [34, 35] and ‘moral distress’ [36–38]. Moral injury ‘is present when (1) there has been a betrayal of what is morally right, (2) by someone who holds legitimate authority (in the military a leader) and (3) in a high-stakes situation’ [39, 40]. Moral injury refers to harm incurred in combat situations, where not only psychiatric but also moral harm is sustained due to people’s experiences [35]. Moral distress emerges ‘when one knows the right course of action, but institutional or cultural constraints prevent one from pursuing it.’ [41, 42]. Harm in case of moral distress is addressed by Thorne et al. They state: ‘The ambiguity and complexity of many NICU cases mean that clinicians may inevitably be left, regardless of the professionalism of their actions, feeling that they may not have done enough, other options could have been followed, errors may have been made’ [37]. According to Thorne et al., a particularly effective way of combating moral distress in the NICU is to target the structural and cultural elements that cause it. MCD can help in this regard [43]. In terms of moral distress, MCD differs in its approach by assuming at the outset that no satisfactory choice is possible, since both alternatives will be accompanied by moral harm [10].

Fourthly, identifying the harm creates a focus on how people are moved by the issue. Baart [44] argues that precisely these emotional responses show what agents find important. Decisions are not made based on general principles, but in concrete situations [45]. Rather than going unacknowledged, emotional bonds should be embraced to allow them to fill their respective agents with love, horror, pain or remorse. The emotions will reveal the proper conceptualisation of what is at stake [46]. According to Rasoal et al. [47], discussing ethical questions in MCD can help them better understand the associated emotions.

A possible downside in this is that emotions can sometimes eclipse the ethical issues, thwarting discussion. Emotions flaring too high could mean that conditions for reflection and dialogue can be hampered [48]. However, reflecting on emotions is important, as they are indications of underlying values [49].

In the fifth place, discussing harm is important in order to acknowledge others’ perspectives on harm. Getting the harm ‘out in the open’ creates understanding and clarity regarding what others in the MCD consider ‘harm’. The various perspectives of the care provider, patient and family on harm are all included in the discussion. Exchanging perspectives as part of MCD is important [50]. This can make people’s views on harm change: by looking at different perspectives, the harm originally perceived by the care providers as a medically pointless procedure for the patient can change into ‘a worthwhile period of time for the patient’.

The first theme identified under the category of ‘dealing with tragedy’ is the importance of acknowledging the harm. Putting the harm into words can help to provide comfort, an effect that has been identified in psychology and trauma processing [51, 52]. Nussbaum arrives at this understanding through her literary and philosophical examination of classic works. For the context of healthcare, this is supported by Vosman and Baart [53]. Baart [44] confirms the importance of devoting time to acknowledging tragedy. He believes it is important for care professionals who are confronted with tragedy to arrive at treatment decisions they can personally take responsibility for and be committed to [54].

Secondly, comfort emerged as one of the resulting themes. Comfort is important, as tragic issues can be associated with feelings of guilt. Nussbaum states: ‘Asking the tragic question requires, first of all, assuming a possible burden of guilt and reparative effort, something people [...] do not always enjoy doing.’ [55]. The inability to comply with both important values but having to make a decision regardless means dealing with a possible burden of guilt and reconciliation with the choices made. According to Nussbaum [55], acknowledging tragedy and confronting the part we ourselves play therein – though the harm may be unavoidable – motivates us to look at what we can do to repair the harm caused by our actions.

This is never entirely achievable, which reveals the tragedy of life itself, which is part of the world in which we live [56–59]. This tragedy is part-and-parcel of our very existence [60] and must be acknowledged [61, 62]. It is important both to look at the residual harm that can be resolved, and at the irreparable, ongoing harm. This fact was reflected in the results, in which several respondents reported devoting greater attention to identifying the long-term harm.

Comfort can also relate to religion or spirituality, a topic which is not explicitly addressed in MCD. Further research is necessary on the potential role of addressing these aspects via MCD.

In the third place, putting harm into words helps professionals to manage tragedy, primarily through reflection on the various perspectives on what constitutes harm. Also, harm can be managed through reflection on the relationship between harm and living a good life. The MCD facilitator may encourage people to be aware that there is no such thing as ‘the perfect life’. Moreover, discussing harm helps by equipping and empowering health professionals with strategies through a shift in perception from what cannot be changed (powerlessness/acceptance) to what can be changed (action). During MCD, the facilitator encourages care professionals to think about what they *can* achieve.

The above-mentioned aspects of managing tragedy can be related to the notion of resilience. Moral resilience is ‘the capacity of an individual to sustain or restore their integrity in response to moral complexity, confusion, distress or setbacks.’ [63]. According to Young and Rushton [64], the emphasis on resilience emerged as a positive response to moral distress. In research among nurses, it has been shown that positive formulations and language are capable of altering the outcomes of situations that are known to cause moral distress [64]. Putting harm into words can contribute towards a positive formulation, if it leads to experience-sharing and an examination of how harm can be mitigated. This technique can inform care professionals how they can manage harm, boosting their resilience.

Fourthly, formulating harm helps ‘weighing up’ through mutual dialogue, which is a significant component of MCD [65]. This is not a simple equation, based on pluses and minuses, but a personal exploration of the important underlying values.

Within the context of public-policy choices, Nussbaum states that cost-benefit issues and issues of tragedy can sometimes become entangled [66]. A cost-benefit analysis aims to uncover ‘a strategy for choice in which weightings are allocated to the available alternative, arriving at some kind of aggregate figure for each major option’ [67]. Such a cost-benefit analysis looks not only at financial benefits, but also at the economic distribution of what is perceived as worthwhile. Tragic questions cannot be answered by a cost-benefit analysis [11]. The consideration is not an addition or subtraction sum, nor is it an economic rationale. Our results support this conceptualisation.

The aim of addressing harm during the MCD discussion is to draw out the underlying key values at play, which helps health professionals to make the necessary choices – choices that are ultimately about what it means to them and their patients to live a good life. Formulating harm not only raises awareness of vulnerabilities and highlights the negatives; it shows that harm also reflects what people hold dear and consider valuable in life [57, 68]. Our results show that discussing harm through dialogue can create shared bonds.

When weighing up the options, the participants in the dialogue look for opportunities for reducing the harm. Nussbaum argues that it is valuable to investigate the basic assumptions underlying the dilemma itself. We should always ask ourselves Hegel’s question: ‘Is there a re-arrangement of our practices that can remove the tragedy?’ [69]. Taking a closer look at the underlying assumptions can sometimes open up new possibilities. But, says Nussbaum: ‘In one way Hegel’s approach to tragedy is too simple. For it ignores the possibility that some degree of tragedy is a structural feature of human life’ [70]. A trace of tragedy may always remain. ‘[...] the residuum of tragedy at the heart of human life. Some rich and complicated aspects of life just are likely to be in tension with some other rich and complicated aspects, and even the wisest Hegelian will not be able to remove the possibility of tragedy [...]’ [68]. We should be aware of the limitations of looking for a way out of the dilemma through the search for a middle ground. Sometimes, harm cannot be resolved or reduced, and this needs attention in MCD.

Finally, formulating harm in MCD can help to repair harm. We encounter the notion of ‘moral repair’ in various contexts: in literature on combat trauma [71], on sexual abuse [72] and on moral distress among nurses [73]. This latter article states that ‘counterstories’ are important in repairing the moral identities of nurses. Telling stories of reliability and nursing expertise are important in order to counterbalance the narrative of nurses as subordinates. Analogously, the narrative element in MCD is important in order to impart strength to one another when dealing with harm. This narrative element is experienced in MCD through the discussion of harm, through the retelling of the case and the joint discussion of the underlying values. Exactly how this narrative element of MCD serves to increase resilience requires further research.

The word ‘repair’ might give the false impression that everything can in the end be controlled in healthcare. As the results show, repair can only be partial; residual harm remains, and not all harm can be repaired. There is sometimes long-term lasting harm that patients must learn to live with, and for which health professionals to a certain extent feel responsible.

## Conclusion

This study showed that addressing harm in moral case deliberation in tragic situations offers an important moral learning opportunity for participants and others involved. Discussing harm reveals what is at stake, and makes visible that tragic decisions have lasting effects. Discussing harm helps to repair negative consequences of decisions where possible. Yet, harm cannot be fully undone.

For health professionals, the added value of addressing harm in MCD lies in the awareness of tragic situations. Through the discussion of harm, MCD contributes to increased sharing, supporting and understanding one another in tragic situations. Also, offering comfort and acknowledgement, and contributing to the resilience of professionals in the difficult decisions they face are benefits of discussing harm in MCD.

## Data Availability

The dataset that supports the conclusion of the article is available in Dutch.

## Abbreviations

CES: Clinical ethics support
MCD: Moral case deliberation
NICU: Neonatal intensive care unit
